# 3,3,6-Tribromo-1-methyl-1*H*-2,1-benzo­thia­zin-4(3*H*)-one 2,2-dioxide

**DOI:** 10.1107/S1600536810045125

**Published:** 2010-11-10

**Authors:** Muhammad Shafiq, Islam Ullah Khan, Muhammad Nadeem Arshad, Ghulam Mustafa

**Affiliations:** a Materials Chemistry Laboratory, Department of Chemistry, GC University, Lahore 54000, Pakistan

## Abstract

In the title compound, C_9_H_6_Br_3_NO_3_S, a halogenated benzothia­zine derivative, the thia­zine ring adopts a sofa conformation. The crystal studied was a racemic twin with a contribution of 72 (1)% of the major domain.

## Related literature

For the synthesis and related structures, see: Shafiq *et al.* (2009*a* ▶,*b* ▶).
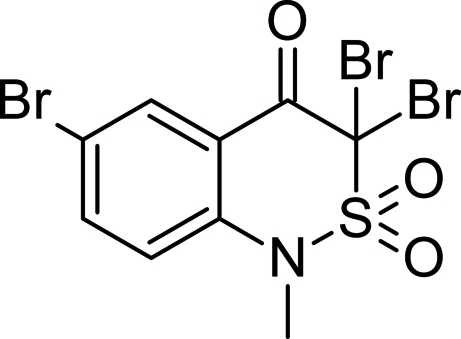


## Experimental

### 

#### Crystal data

C_9_H_6_Br_3_NO_3_S
*M* *_r_* = 447.94
Orthorhombic, 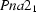

*a* = 14.922 (1) Å
*b* = 12.1310 (8) Å
*c* = 7.0811 (4) Å
*V* = 1281.81 (14) Å^3^
*Z* = 4
Mo *K*α radiation
μ = 9.60 mm^−1^
*T* = 296 K
0.28 × 0.21 × 0.12 mm

#### Data collection

Bruker Kappa APEXII CCD diffractometer
Absorption correction: multi-scan (*SADABS*; Bruker, 2007 ▶) *T*_min_ = 0.096, *T*_max_ = 0.144
7900 measured reflections
2941 independent reflections
2221 reflections with *I* > 2σ(*I*)
*R*_int_ = 0.033

#### Refinement

*R*[*F* ^2^ > 2σ(*F* ^2^)] = 0.035
*wR*(*F* ^2^) = 0.071
*S* = 0.99
2941 reflections
156 parameters
1 restraint
H-atom parameters constrained
Δρ_max_ = 0.54 e Å^−3^
Δρ_min_ = −0.55 e Å^−3^
Absolute structure: Flack (1983 ▶), 1242 Friedel pairs
Flack parameter: 0.00 (3)

### 

Data collection: *APEX2* (Bruker, 2007 ▶); cell refinement: *SAINT* (Bruker, 2007 ▶); data reduction: *SAINT*; program(s) used to solve structure: *SHELXS97* (Sheldrick, 2008 ▶); program(s) used to refine structure: *SHELXL97* (Sheldrick, 2008 ▶); molecular graphics: *ORTEP-3 for Windows* (Farrugia, 1997 ▶) and *PLATON* (Spek, 2009 ▶); software used to prepare material for publication: *WinGX* (Farrugia, 1999 ▶) and *PLATON*.

## Supplementary Material

Crystal structure: contains datablocks I, global. DOI: 10.1107/S1600536810045125/bt5404sup1.cif

Structure factors: contains datablocks I. DOI: 10.1107/S1600536810045125/bt5404Isup2.hkl

Additional supplementary materials:  crystallographic information; 3D view; checkCIF report

## Figures and Tables

**Fig. 1.**
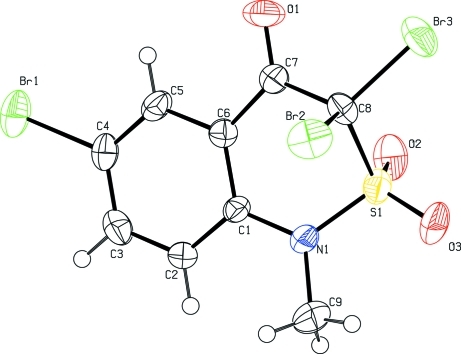
Perspective view of the title compound with displacement ellipsoids drawn at the 50% probability level.

## supplementary crystallographic information

### Comment

The title compound, (I), is structurally related to the already reported crystal structures of 3,3-dichloro-1-ethyl-1*H*-2,1-benzothiazin-4 (3*H*)-one 2,2-dioxide, (II), (Shafiq *et al.*, 2009*a*) and 6-bromo-3,3-dichloro-1-methyl-1*H*-2,1-benzothiazin-4 (3*H*)-one 2,2-dioxide, (III), (Shafiq *et al.*, 2009*b*).

Like (II) and (III) the thiazine (C1/C6/C7/C8/S1/N1) ring in the crystal structure adopted a sofa form.

### Experimental

The title compound was prepared following the already reported procedure (Shafiq *et al.*, 2009*b*).

### Refinement

All  H-atoms were positioned with idealized geometry with C—H = 0.93 Å and C—H = 0.96 Å and were refined using a riding model with *U*_iso_(H) = 1.2 *U*_eq_(C) or *U*_iso_(H) = 1.5 *U*_eq_(C_methyl_). The crystal turned out to be a racemic twin with a contribution of 72 (1)% of the major domain.

### Crystal data

**Table:**

C_9_H_6_Br_3_NO_3_S	*F*(000) = 848
*M**_r_* = 447.94	*D*_x_ = 2.321 Mg m^−^^3^
Orthorhombic, *P**n**a*2_1_	Mo *K*α radiation, λ = 0.71073 Å
Hall symbol: P 2c -2n	Cell parameters from 2326 reflections
*a* = 14.922 (1) Å	θ = 3.3–24.8°
*b* = 12.1310 (8) Å	µ = 9.60 mm^−^^1^
*c* = 7.0811 (4) Å	*T* = 296 K
*V* = 1281.81 (14) Å^3^	Needle, light brown
*Z* = 4	0.28 × 0.21 × 0.12 mm

### Data collection

**Table:**

Bruker Kappa APEXII CCD diffractometer	2941 independent reflections
Radiation source: fine-focus sealed tube	2221 reflections with *I* > 2σ(*I*)
graphite	*R*_int_ = 0.033
φ and ω scans	θ_max_ = 28.2°, θ_min_ = 2.7°
Absorption correction: multi-scan (*SADABS*; Bruker, 2007)	*h* = −15→19
*T*_min_ = 0.096, *T*_max_ = 0.144	*k* = −16→10
7900 measured reflections	*l* = −8→9

### Refinement

**Table:**

Refinement on *F*^2^	Secondary atom site location: difference Fourier map
Least-squares matrix: full	Hydrogen site location: inferred from neighbouring sites
*R*[*F*^2^ > 2σ(*F*^2^)] = 0.035	H-atom parameters constrained
*wR*(*F*^2^) = 0.071	*w* = 1/[σ^2^(*F*_o_^2^) + (0.0309*P*)^2^] where *P* = (*F*_o_^2^ + 2*F*_c_^2^)/3
*S* = 0.99	(Δ/σ)_max_ = 0.001
2941 reflections	Δρ_max_ = 0.54 e Å^−^^3^
156 parameters	Δρ_min_ = −0.55 e Å^−^^3^
1 restraint	Absolute structure: Flack (1983), 1242 Friedel pairs
Primary atom site location: structure-invariant direct methods	Flack parameter: 0.00 (3)

### Special details

**Table:**

Geometry. All s.u.'s (except the s.u. in the dihedral angle between two l.s. planes) are estimated using the full covariance matrix. The cell s.u.'s are taken into account individually in the estimation of s.u.'s in distances, angles and torsion angles; correlations between s.u.'s in cell parameters are only used when they are defined by crystal symmetry. An approximate (isotropic) treatment of cell s.u.'s is used for estimating s.u.'s involving l.s. planes.
Refinement. Refinement of *F*^2^ against ALL reflections. The weighted *R*-factor *wR* and goodness of fit *S* are based on *F*^2^, conventional *R*-factors *R* are based on *F*, with *F* set to zero for negative *F*^2^. The threshold expression of *F*^2^ > σ(*F*^2^) is used only for calculating *R*-factors(gt) *etc*. and is not relevant to the choice of reflections for refinement. *R*-factors based on *F*^2^ are statistically about twice as large as those based on *F*, and *R*- factors based on ALL data will be even larger.

### Fractional atomic coordinates and isotropic or equivalent isotropic displacement parameters (Å^2^)

**Table:**

	*x*	*y*	*z*	*U*_iso_*/*U*_eq_	
Br1	0.56408 (4)	0.70021 (5)	0.24956 (8)	0.05225 (17)	
S1	0.21026 (9)	0.32623 (11)	0.3603 (2)	0.0368 (3)	
O1	0.2102 (3)	0.6272 (3)	0.2328 (8)	0.0631 (13)	
N1	0.3103 (2)	0.3077 (3)	0.2744 (6)	0.0341 (9)	
C1	0.3690 (3)	0.3998 (3)	0.2654 (7)	0.0269 (10)	
Br2	0.17819 (4)	0.39549 (5)	−0.05254 (9)	0.05073 (17)	
O2	0.2124 (3)	0.3737 (3)	0.5431 (5)	0.0505 (11)	
C2	0.4609 (3)	0.3843 (3)	0.2690 (8)	0.0352 (11)	
H2	0.4841	0.3132	0.2754	0.042*	
Br3	0.05403 (4)	0.47720 (5)	0.28558 (9)	0.05946 (19)	
O3	0.1596 (3)	0.2288 (3)	0.3279 (6)	0.0569 (11)	
C3	0.5178 (3)	0.4721 (4)	0.2632 (8)	0.0385 (11)	
H3	0.5794	0.4605	0.2657	0.046*	
C4	0.4844 (3)	0.5789 (4)	0.2536 (7)	0.0346 (11)	
C5	0.3947 (3)	0.5960 (3)	0.2469 (7)	0.0333 (11)	
H5	0.3727	0.6676	0.2400	0.040*	
C6	0.3348 (3)	0.5074 (3)	0.2503 (7)	0.0279 (10)	
C7	0.2389 (3)	0.5345 (4)	0.2336 (6)	0.0309 (10)	
C8	0.1729 (3)	0.4378 (4)	0.2090 (6)	0.0325 (11)	
C9	0.3419 (4)	0.1959 (4)	0.2311 (10)	0.0553 (16)	
H9C	0.3840	0.1989	0.1287	0.083*	
H9B	0.2919	0.1506	0.1958	0.083*	
H9A	0.3705	0.1652	0.3405	0.083*	

### Atomic displacement parameters (Å^2^)

**Table:**

	*U*^11^	*U*^22^	*U*^33^	*U*^12^	*U*^13^	*U*^23^
Br1	0.0503 (4)	0.0549 (3)	0.0516 (3)	−0.0277 (3)	0.0009 (3)	−0.0028 (3)
S1	0.0342 (8)	0.0388 (7)	0.0376 (6)	−0.0059 (6)	0.0022 (6)	0.0082 (6)
O1	0.039 (2)	0.0306 (19)	0.120 (4)	0.0096 (17)	−0.002 (3)	−0.006 (3)
N1	0.029 (2)	0.0234 (19)	0.050 (3)	−0.0030 (16)	0.001 (2)	0.002 (2)
C1	0.031 (3)	0.022 (2)	0.027 (2)	0.0008 (18)	0.004 (2)	0.004 (2)
Br2	0.0663 (4)	0.0518 (3)	0.0341 (2)	0.0025 (3)	−0.0111 (3)	−0.0066 (3)
O2	0.053 (3)	0.069 (3)	0.029 (2)	−0.009 (2)	0.0066 (18)	0.0041 (18)
C2	0.029 (3)	0.029 (2)	0.048 (3)	0.0052 (19)	0.005 (3)	0.007 (3)
Br3	0.0275 (3)	0.0677 (4)	0.0831 (5)	0.0025 (3)	0.0073 (3)	−0.0042 (4)
O3	0.047 (2)	0.043 (2)	0.081 (3)	−0.0203 (19)	0.005 (2)	0.009 (2)
C3	0.025 (3)	0.049 (3)	0.041 (3)	0.000 (2)	0.002 (3)	0.002 (3)
C4	0.031 (3)	0.039 (3)	0.033 (2)	−0.012 (2)	0.000 (2)	0.000 (3)
C5	0.041 (3)	0.022 (2)	0.037 (3)	−0.0056 (19)	0.001 (2)	−0.002 (2)
C6	0.029 (3)	0.028 (2)	0.027 (2)	−0.0008 (18)	0.000 (2)	−0.001 (2)
C7	0.030 (3)	0.028 (2)	0.034 (2)	−0.001 (2)	0.000 (2)	−0.001 (2)
C8	0.025 (3)	0.039 (3)	0.033 (3)	0.000 (2)	0.001 (2)	−0.007 (2)
C9	0.063 (4)	0.027 (3)	0.076 (4)	−0.002 (3)	0.014 (4)	−0.006 (3)

### Geometric parameters (Å, °)

**Table:**

Br1—C4	1.892 (4)	C2—H2	0.9300
S1—O2	1.417 (4)	Br3—C8	1.916 (5)
S1—O3	1.422 (4)	C3—C4	1.390 (6)
S1—N1	1.627 (4)	C3—H3	0.9300
S1—C8	1.814 (5)	C4—C5	1.356 (7)
O1—C7	1.204 (5)	C5—C6	1.398 (6)
N1—C1	1.421 (5)	C5—H5	0.9300
N1—C9	1.469 (6)	C6—C7	1.473 (6)
C1—C2	1.384 (6)	C7—C8	1.542 (6)
C1—C6	1.406 (5)	C9—H9C	0.9600
Br2—C8	1.923 (4)	C9—H9B	0.9600
C2—C3	1.362 (6)	C9—H9A	0.9600
			
O2—S1—O3	119.9 (2)	C4—C5—C6	120.9 (4)
O2—S1—N1	112.1 (2)	C4—C5—H5	119.6
O3—S1—N1	108.2 (2)	C6—C5—H5	119.6
O2—S1—C8	104.1 (2)	C5—C6—C1	118.9 (4)
O3—S1—C8	111.2 (2)	C5—C6—C7	116.6 (4)
N1—S1—C8	99.4 (2)	C1—C6—C7	124.5 (4)
C1—N1—C9	121.2 (4)	O1—C7—C6	123.7 (4)
C1—N1—S1	118.3 (3)	O1—C7—C8	118.9 (4)
C9—N1—S1	120.0 (3)	C6—C7—C8	117.4 (4)
C2—C1—C6	119.2 (4)	C7—C8—S1	107.7 (3)
C2—C1—N1	120.3 (4)	C7—C8—Br3	111.7 (3)
C6—C1—N1	120.6 (4)	S1—C8—Br3	107.7 (2)
C3—C2—C1	120.7 (4)	C7—C8—Br2	106.6 (3)
C3—C2—H2	119.6	S1—C8—Br2	110.9 (2)
C1—C2—H2	119.6	Br3—C8—Br2	112.2 (2)
C2—C3—C4	120.5 (4)	N1—C9—H9C	109.5
C2—C3—H3	119.8	N1—C9—H9B	109.5
C4—C3—H3	119.8	H9C—C9—H9B	109.5
C5—C4—C3	119.9 (4)	N1—C9—H9A	109.5
C5—C4—Br1	120.1 (4)	H9C—C9—H9A	109.5
C3—C4—Br1	120.0 (4)	H9B—C9—H9A	109.5
			
O2—S1—N1—C1	−54.7 (4)	C2—C1—C6—C7	176.2 (5)
O3—S1—N1—C1	170.9 (4)	N1—C1—C6—C7	−3.0 (7)
C8—S1—N1—C1	54.8 (4)	C5—C6—C7—O1	−6.2 (8)
O2—S1—N1—C9	117.5 (5)	C1—C6—C7—O1	175.1 (5)
O3—S1—N1—C9	−16.9 (5)	C5—C6—C7—C8	171.7 (4)
C8—S1—N1—C9	−133.0 (5)	C1—C6—C7—C8	−7.1 (7)
C9—N1—C1—C2	−18.4 (7)	O1—C7—C8—S1	−142.5 (5)
S1—N1—C1—C2	153.6 (4)	C6—C7—C8—S1	39.5 (5)
C9—N1—C1—C6	160.7 (5)	O1—C7—C8—Br3	−24.5 (6)
S1—N1—C1—C6	−27.2 (6)	C6—C7—C8—Br3	157.6 (3)
C6—C1—C2—C3	1.8 (8)	O1—C7—C8—Br2	98.3 (5)
N1—C1—C2—C3	−179.1 (5)	C6—C7—C8—Br2	−79.6 (4)
C1—C2—C3—C4	0.0 (8)	O2—S1—C8—C7	57.1 (4)
C2—C3—C4—C5	−1.1 (8)	O3—S1—C8—C7	−172.5 (3)
C2—C3—C4—Br1	179.0 (4)	N1—S1—C8—C7	−58.7 (3)
C3—C4—C5—C6	0.3 (8)	O2—S1—C8—Br3	−63.5 (3)
Br1—C4—C5—C6	−179.8 (4)	O3—S1—C8—Br3	66.9 (3)
C4—C5—C6—C1	1.6 (7)	N1—S1—C8—Br3	−179.3 (2)
C4—C5—C6—C7	−177.3 (4)	O2—S1—C8—Br2	173.4 (3)
C2—C1—C6—C5	−2.6 (7)	O3—S1—C8—Br2	−56.2 (3)
N1—C1—C6—C5	178.3 (4)	N1—S1—C8—Br2	57.6 (3)
